# A deep learning approach with subregion partition in MRI image analysis for metastatic brain tumor

**DOI:** 10.3389/fninf.2022.973698

**Published:** 2022-08-03

**Authors:** Jiaxin Shi, Zilong Zhao, Tao Jiang, Hua Ai, Jiani Liu, Xinpu Chen, Yahong Luo, Huijie Fan, Xiran Jiang

**Affiliations:** ^1^Department of Biomedical Engineering, School of Intelligent Medicine, China Medical University, Shenyang, China; ^2^Department of Neurosurgery, The First Affiliated Hospital of China Medical University, Shenyang, China; ^3^Department of Radiology, Cancer Hospital of China Medical University, Liaoning Cancer Hospital and Institute, Shenyang, China; ^4^State Key Laboratory of Robotics, Shenyang Institute of Automation, Chinese Academy of Sciences, Shenyang, China

**Keywords:** deep learning, brain metastasis, MRI, NSCLC, breast cancer

## Abstract

**Purpose:**

To propose a deep learning network with subregion partition for predicting metastatic origins and EGFR/HER2 status in patients with brain metastasis.

**Methods:**

We retrospectively enrolled 140 patients with clinico-pathologically confirmed brain metastasis originated from primary NSCLC (*n* = 60), breast cancer (BC, *n* = 60) and other tumor types (*n* = 20). All patients underwent contrast-enhanced brain MRI scans. The brain metastasis was subdivided into phenotypically consistent subregions using patient-level and population-level clustering. A residual network with a global average pooling layer (RN-GAP) was proposed to calculate deep learning-based features. Features from each subregion were selected with least absolute shrinkage and selection operator (LASSO) to build logistic regression models (LRs) for predicting primary tumor types (LR-NSCLC for the NSCLC origin and LR-BC for the BC origin), EGFR mutation status (LR-EGFR) and HER2 status (LR-HER2).

**Results:**

The brain metastasis can be partitioned into a marginal subregion (S1) and an inner subregion (S2) in the MRI image. The developed models showed good predictive performance in the training (AUCs, LR-NSCLC vs. LR-BC vs. LR-EGFR vs. LR-HER2, 0.860 vs. 0.909 vs. 0.850 vs. 0.900) and validation (AUCs, LR-NSCLC vs. LR-BC vs. LR-EGFR vs. LR-HER2, 0.819 vs. 0.872 vs. 0.750 vs. 0.830) set.

**Conclusion:**

Our proposed deep learning network with subregion partitions can accurately predict metastatic origins and EGFR/HER2 status of brain metastasis, and hence may have the potential to be non-invasive and preoperative new markers for guiding personalized treatment plans in patients with brain metastasis.

## Introduction

Brain metastasis is a common complication in patients with malignant tumors and represent one of the most frequent neurological complications of systemic cancer with incidence rates of 20–50% depending on the type of primary tumor (Brastianos et al., 2015; Cagney et al., 2017). In clinical practice, the presence of brain metastasis is much more common than primary brain tumors, and has been a major cause of morbidity and mortality (Achrol et al., 2019). In recent years, the incidence of brain metastasis has increased because of advances in neuroimaging procedures (Hardesty and Nakaji, 2016). Among different types of primary tumors, non-small cell lung cancer (NSCLC, 41–56%) and breast cancer (BC, 15–30%) are the most common origins of brain metastasis (Bekaert et al., 2017). Early and accurate identification of the brain metastatic origin before surgery could change the personal treatment plan, and hence of great clinical significance (Kniep et al., 2019).

For NSCLC patients with brain metastasis, the epidermal growth factor receptor (EGFR*)* gene mutation status is essential for treatment strategies, such as EGFR-tyrosine kinase inhibitors (EGFR-TKIs) therapy (Guo et al., 2020). For BC patients with brain metastasis, the HER2 status is crucial for selecting therapeutic methods (Loibl and Gianni, 2017). This is because patients with HER2 positive are routinely treated with targeted antibody therapy (Koboldt et al., 2012; Lam et al., 2014) and usually have a poor prognosis (Cameron et al., 2017). Considering tissue acquisition from the primary tumor is not always clinically possible, the metastases can be an important alternate to reflect the characteristics and gene status of the primary tumor (Krawczyk et al., 2014). However, radiologists can hardly assess metastatic origins or gene status of primary tumor through visual examination on MRI data due to the absence of specific markers (Kniep et al., 2019).

Advances in computer-aided diagnosis (CAD) and artificial intelligence have played an increasingly important role in the field of medical imaging (Gore, 2020; Barragán-Montero et al., 2021). Radiomics, as an emerging field refers to systematic calculate and analysis big amount of quantitative features from medical imaging (Lambin et al., 2017). However, there were limited studies reported CAD-based identification of the metastatic origin of brain metastasis (Ortiz-Ramon et al., 2017; Béresová et al., 2018). Previous reports both assumed that the brain metastasis is homogeneous throughout the tumor volume. However, recent researches demonstrated that solid tumors are heterogeneous with some tumor regions are more biologically aggressive and may reflect different biological processes (Gatenby et al., 2013). This was known as intratumor heterogeneity (ITH), and was indicated to have significant implications that represent distinct tumor progression (O’Connor et al., 2015). Subregion-based radiomics algorithms were suggested to divide the whole tumor area into intratumor subregions, and hence allow to capture valuable information from the subregions (Lin et al., 2021). To data, subregion radiomics analyses have been conducted in breast cancer (Fan et al., 2018), lung cancer (Shen et al., 2021) and esophageal squamous (Xie et al., 2019), and demonstrated to significantly improve the diagnostic performance of radiomics methods. To our knowledge, subregion radiomics has not been investigated in brain metastasis.

In this study, we subdivided the brain metastasis into phenotypically consistent subregions based on patient- and population-level clustering, and evaluated handcrafted features and deep learning-based features from a proposed residual network with a global average pooling layer (RN-GAP) for predicting metastatic origins of brain metastasis and assessing EGFR and HER2 status.

## Materials and methods

### Patients

We included 140 patients harboring brain metastasis from January 2017 to September 2020. The patients met the following criteria (i) with the diagnosis of brain metastasis confirmed by pathological examination; (ii) 3.0T brain MRI scan before treatment; and (iii) complete records of the EGFR gene mutation status by gene sequencing. The exclusion criteria included: (i) not received any anti-cancer treatment (including radiotherapy, chemotherapy or biotherapy) before the MRI examination; (ii) poor image quality or severe motion artifacts; and (iii) incomplete clinical information. To determine EGFR mutation status for the patients, genetic sequencing analysis was performed based puncture biopsy of the primary tumor before treatment. To determine HER2 status, immunohistochemistry (IHC) and fluorescence in situ hybridization (FISH) analysis were used (Ruan et al., 2017; Fehrenbacher et al., 2020).

### MRI scans and tumor segmentation

For each patient, the T1CE MR were obtained with a 3.0T MRI scanner (Siemens Magnetom Verioio, Erlangen, Germany). The GA-DTPA was used as contrast agent when performing the T1CE MRI scanning. The parameters of the T1CE MRI sequence were shown in Table 1. To obtain the whole tumor region, a radiologist with 4 years of experience manually outlined edges of the whole tumor region layer by layer. The segmented region of interest (ROI) was stored in a.NII format and used for computer-aided analyses.

**TABLE 1:** Parameters of the brain MRI screenings.

Parameters	MRI
Repetition time/echo time (TR/TE) (ms)	120/2.48
Slice thickness (mm)	5
Spacing between slices (mm)	6.5
Acquisition matrix	320 × 240
Pixel spacing (mm)	0.359 × 0.359
Field of view (mm)	600 × 640

### Intratumor partition

We used a three-step approach for intratumor partition of the brain metastasis. Firstly, MRI local entropy was calculated for each patient’s slice using a 9 × 9 neighborhood. Secondly, Patient-level clustering was then applied to partition the tumor regions into many superpixels based on the calculated local entropy maps and MRI pixel intensity to represent the spatial heterogeneity (Wu et al., 2016). K-means clustering (Kanungo et al., 2002) was performed using the Euclidean distance between pixel intensity and entropy intensity as a distance metric. In each ROI, pixels with similar pixel intensities and entropy intensities were clustered into many superpixels by patient-level clustering based on the principle of K-means clustering. Finally, superpixels from all patients were gathered to perform the population-level clustering via exploring the similarity of inter- and intra-patients using hierarchical clustering (Johnson, 1967). The number of partitioned subregions (clusters k) was set from 2 to 10 as suggested (Pham et al., 2005). To determine the optimal number of partitioned subregions, the Calinski-Harabasz (CH) index was used as a criterion according to a previous work (Caliński and Harabasz, 1974). Differences in pixel intensity and entropy intensity of metastatic origins and gene status were evaluated with *t*-test.

### Deep learning network development

**FIGURE 1:**
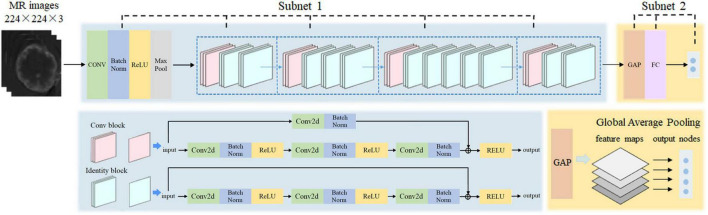
Our deep learning network architecture: adding a global average pooling layer based on the residual network.

We proposed a RN-GAP to extract deep learning-based radiomics features from the partitioned subregions of brain metastasis in MRI images. The network architecture of ResNet50 was shown in Supplementary Table 1 (Brito et al., 2019). Figure 1 showed the architecture of the RN-GAP network. We used 14 million natural images from ImageNet database (Deng et al., 2009) to pre-train the ResNet50 network as Subnet 1. The Subnet 2 was composed of a GAP layer (Lin et al., 2013), a fully connected layer and a softmax output layer. Global averaging pooling (GAP) layer calculated the average value of each feature map as an output node and forms all output nodes into a feature vector, reducing number of parameters. To avoid overfitting, the GAP layer was used before the fully connected layer with 512 neurons, which was followed by a softmax output layer. To fit the RN-GAP’s architecture, MRI images of the largest ROIs in the partitioned subregions were resized to 224 × 224, and the unnecessary background was removed. The proposed RN-GAP network was constructed based on the Python v3.6 platform.

### Radiomics handcrafted and deep learning-based feature calculation

Before extraction of radiomics features, pre-processing of MR images were performed with detailed descriptions can be found in Supplementary Table 2. We applied the open source package Pyradiomics^1^ based on the Python 3.6 platform to calculate handcrafted radiomics features from brain MRI images according to reported protocols (van Griethuysen et al., 2017).

To obtain deep learning-based radiomics features, MRI images of the largest ROIs in the segmented subregions of brain metastasis were used to feed the RN-GAP. Values of the 512 neurons from the fully connected layer were recorded and used as deep learning-based features for further analysis.

### Feature selection and logistic regression model construction

**FIGURE 2:**
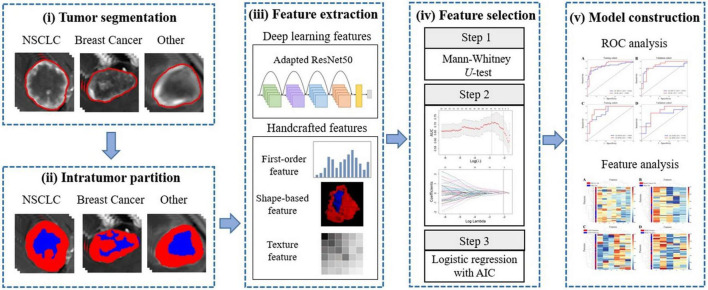
The flowchart of radiomics analyses.

Inter and intra-observer reproducibilities of all features was assessed by inter-class and intar-class correlation coefficient (ICC) analyses (Leijenaar et al., 2013). Then, the Mann-Whitney *U*-test was performed to further select features with *P* < 0.05 as predictors with significant differences. Finally, the least absolute shrinkage and selection operator (LASSO) algorithm was used for dimensionality reduction of the calculated features with 10-fold cross-validation. The features were substituted into multivariate logistic regression analysis using the stepwise regression method to obtain the respective regression coefficients of the most predictive features. The feature selecting method was applied to both handcrafted features and deep learning-based features. Coefficients of the selected features were weighted to construct logistic regression models (LRs) for predicting metastatic origins (LR-NSCLC and LR-BC), EGFR mutation status (LR-EGFR) and HER2 status (LR-HER2). Figure 2 showed the flowchart of this study.

## Results

### Patient characteristics

We finally included 140 patients in the study, 41 males and 99 females, aged 29–77 years. There were 60, 60, and 20 patients harboring brain metastases from primary NSCLC, breast cancer and other types of malignant tumors, respectively. The clinical characteristics of the enrolled patients were shown in Table 2.

**TABLE 2:** Clinical characteristics of the enrolled patients.

Tumor type	Gene status	Gender		Age (Mean ± SD)
		Female	Male	
NSCLC		30	30	57.82 ± 7.26
	EGFR mutant	20	10	56.67 ± 7.20
	EGFR wild-type	10	20	58.97 ± 7.13
Breast cancer		60	0	53.63 ± 10.53
	HER2 positive	30	0	54.03 ± 10.82
	HER2 negative	30	0	53.23 ± 10.21
Other		9	11	53.23 ± 10.21
Total		99	41	58.80 ± 13.45

NCSLC, Non-small-cell lung cancer.

### Intratumor partition

**FIGURE 3:**
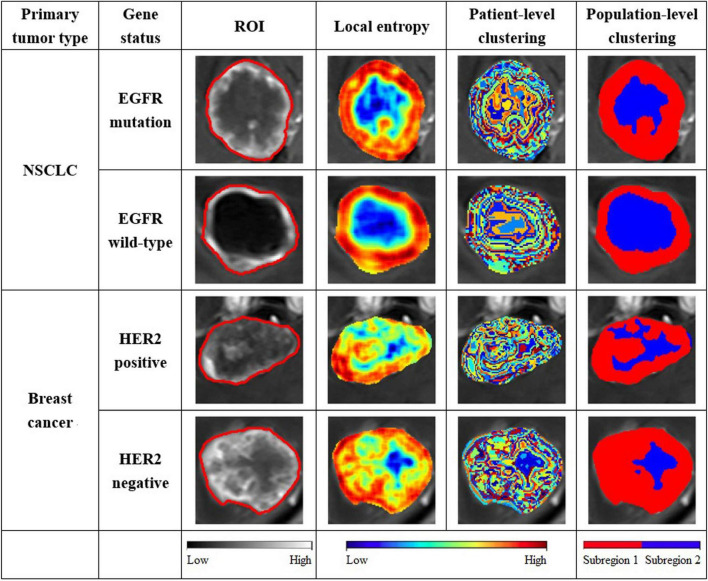
Intratumoral partitions in the MRI images of patients with brain metastasis. The third column represents ROIs in the MRI image. The fourth column represents local entropy maps within the tumor. The fifth column represents the results of patient-level clustering. The sixth column represents the results of population-level clustering.

**FIGURE 4:**
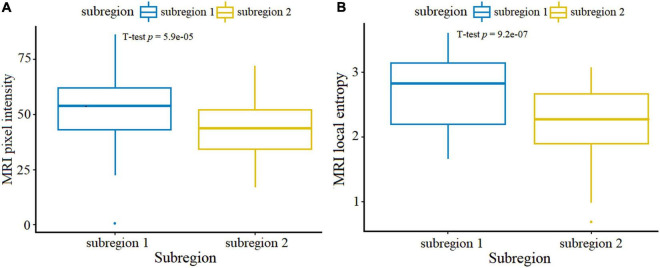
Boxplots showed MRI pixcel intensity **(A)** and local entropy **(B)** between the partitioned S1 and S2 in all patients. *P*-values were obtained using the *t*-test.

Figure 3 shows results of intratumoral partitions in the MRI images of brain metastasis using a patient- and population-level clustering approach. The brain metastasis can be divided into two subregions, which were denoted as S1 (marginal subregion) and S2 (inner subregion). There were obvious differences between S1 and S2. Supplementary Figure 1 showed different CH values when the clusters k was set from 2 to 10. The optimal CH index was calculated to decide the number of intratumoral subregions when k equals to 2. Figure 4 indicated that the distribution of MRI pixel intensity and local entropy of the S1 and S2 were significantly different. Compared with S1 and S2, S1 was more associated with both MRI pixel intensity and local entropy. This indicates a higher degree of tumor heterogeneity in S1.

### Development and evaluation of logistic regression models

All handcrafted and deep learning-based features were extracted from the partationed subregions and selected to develop logistic regression models for predicting brain metastasis originated from primary NSCLC (LR-NSCLC) and breast cancer (LR-BC), and for predicting the EGFR mutation (LR-EGFR) status and HER2 (LR-HER2) status. Formulas for each model were shown below:

LR-NSCLC = – 1.1569 + 0.2105 × wavelet-HLH_glrlm_LongRunEmphasis – 1.4567 × wavelet-LHL_glszm_SmallAreaHighGrayLevelEmphasis – 1.2819 × wavelet-HHH_firstorder_RootMeanSquared + 0.2768 × wavelet-HHH_glrlm_LongRunLowGrayLevelEmphasis + 0.9681 × lbp-3D-k_firstorder_Mean – 0.5264 × DL_215 – 0.8798 × DL_400

LR-BC = – 1.5412 + 1.9088 × wavelet-HHL_ glcm_InverseVariance + 1.9784 × wavelet-HHH_ firstorder_InterquartileRange – 0.9107 × DL_317 – 1.0072 × DL_144 – 0.6121 × DL_122

LR-EGFR = 0.03461 + 1.8998 × squareroot_glszm_Small AreaHighGrayLevelEmphasi –0.8220 × log-sigma-5-mm-3D_ngtdm_Contrast + 1.6274 × wavelet-LHL_ firstorder_Skewness + 0.9110 × lbp-3D-k_firstorder_ MeanAbsoluteDeviation + 1.1375 × lbp-3D-m2_glszm_ GrayLevelVariance + 0.0129 × DL_336

LR-HER2 = – 0.2327 + 0.5867 × wavelet-LLH_ firstorder_10Percentile – 5.5909 × square_ngtdm_Busyness – 1.5990 × DL_317 + 2.4984 × wavelet-HHL_ glcm_Imc1 – 0.2158 × log-sigma-3-mm-3D_glszm _LargeAreaHighGrayLevelEmphasis.

**FIGURE 5:**
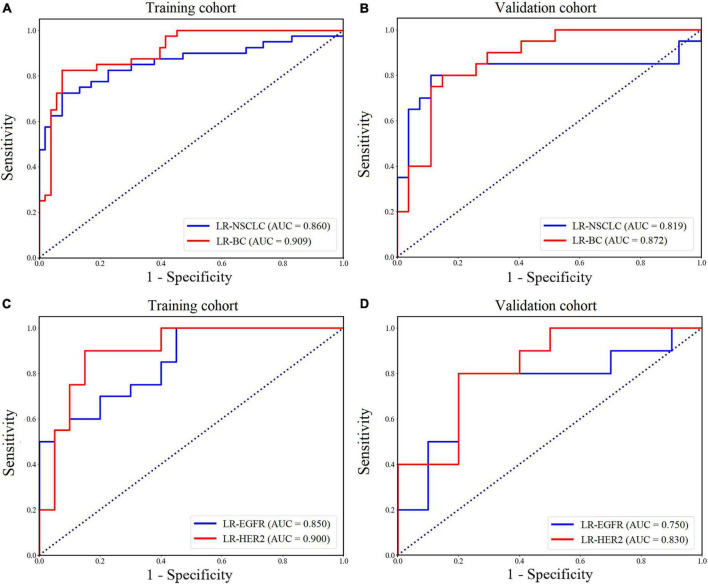
Receiver operating characteristic (ROC) curves of the models for predicting metastatic origins **(A,B)** in the training **(A)** and validation **(B)** cohort, and for predicting the EGFR mutation and HER2 status **(C,D)** in the training **(C)** and validation **(D)** cohort.

Table 3 compared prediction performance of the established LRs. Among the models, the LR-EGFR generated the lowest AUCs in both training and validation cohorts. Figure 5 showed ROC curves of the LR-NSCLC, LR-BC, LR-EGFR and LR-HER2.

**TABLE 3:** Prediction performance of the established logistic regression models.

LR model	Feature size	Cohort	AUC (95%CI)	ACC	SPE	SEN
LR-NSCLC	7	Training	0.860 (0.775–0.944)	0.796	0.925	0.725
		Validation	0.819 (0.665–0.972)	0.809	0.889	0.800
LR-BC	5	Training	0.909 (0.850–0.969)	0.839	0.925	0.825
		Validation	0.872 (0.771–0.973)	0.787	0.852	0.800
LR-EGFR	6	Training	0.850 (0.735–0.965)	0.700	0.550	1.000
		Validation	0.750 (0.514–0.987)	0.700	0.800	0.800
LR-HER2	5	Training	0.900 (0.800–1.000)	0.850	0.850	0.900
		Validation	0.830 (0.645–1.000)	0.750	0.800	0.800

### Radiomics feature analysis

**FIGURE 6:**
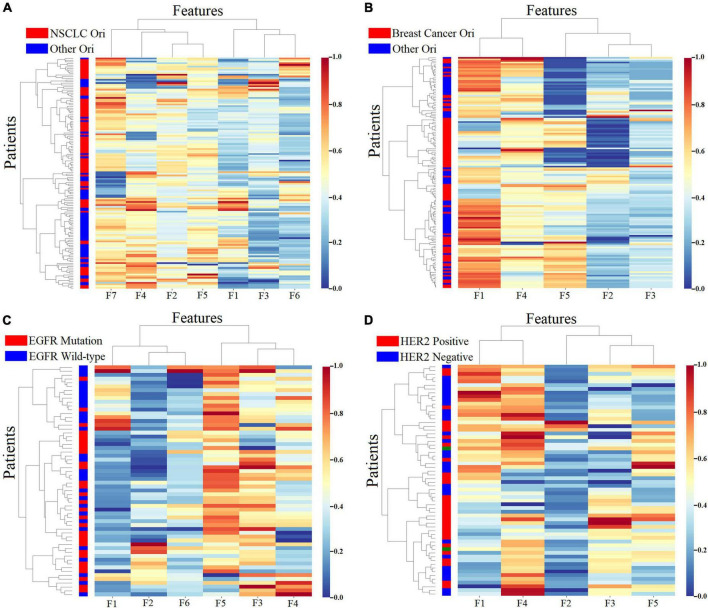
Unsupervised cluster analysis of the selected features for predicting the NSCLC originated metastasis **(A)**, breast cancer originated metastasis **(B)**, EGFR mutation status **(C)** and HER2 status **(D)**. The *x*-axis represents the selected features with the highest predictive values. The *y*-axis represents all patients with brain metastasis.

The optimal radiomics features were identified from both handcrafted and deep learning-based features from brain metastasis subregions. For predicting brain metastasis originated from primary NSCLC, seven features were selected, five were handcrafted features and two were deep learning-based features. For predicting the breast cancer origin, five features were selected, two were handcrafted features and three were deep learning-based features. For predicting the EGFR mutation status, six features were selected, five were handcrafted features and one was deep learning feature. For predicting the HER2 status, five features were selected, four were handcrafted features and one was deep learning feature. As shown in Table 4, all features were obtained from S1. Figure 6 depicted results of the unsupervised cluster analysis of the selected features for all patients, which indicated similarities and differences of features across patients.

**TABLE 4:** Prediction performance of the selected features.

Feature	Mean ± SD				AUC	*P*	ICC (inter–)	ICC (intra–)
	NSCLC	BC	EGFR	HER2				
Wavelet-HLH_glrlm_LongRunEmphasis (F1)	–0.003 ± 0.670	–	–	–	0.594	0.175	0.875	0.864
Wavelet-LHL_glszm_SmallAreaHighGrayLevel Emphasis (F2)	–0.004 ± 0.658	–	–	–	0.753	< 0.001	0.896	0.881
Wavelet-HHH_firstorder_RootMeanSquared (F3)	–0.344 ± 0.642	–	–	–	0767	< 0.001	0.877	0.869
Wavelet-HHH_glrlm_LongRunLowGrayLevel Emphasis (F4)	0.302 ± 0.817	–	–	–	0.617	< 0.134	0.884	0.873
Lbp-3D-k_firstorder_Mean (F5)	0.145 ± 0.689	–	–	–	0.658	0.007	0.868	0.882
DL_215 (F6)	0.035 ± 0.789	–	–	–	0.571	0.106	0.899	0.895
DL_400 (F7)	–0.050 ± 0.896	–	–	–	0.628	0.001	0.852	0.868
Wavelet-HHL_glcm_InverseVariance (F1)	–	0.202 ± 0.987	–	–	0.725	< 0.001	0.872	0.856
Wavelet-HHH_firstorder_InterquartileRange (F2)	–	0.214 ± 0.972	–	–	0.718	< 0.001	0.861	0.858
DL_317 (F3)	–	0.111 ± 1.114	–	–	0.625	0.004	0.863	0.870
DL_144 (F4)	–	0.073 ± 1.079	–	–	0.661	0.017	0.860	0889
DL_122 (F5)	–	0.080 ± 1.001	–	–	0.636	0.010	0.869	0.898
Squareroot_glszm_SmallAreaHighGrayLevel Emphasis (F1)	–		–0.102 ± 0.617	–	0.648	0.008	0.855	0.874
Log-sigma-5-mm.3D_ngtdm_Contrast (F2)	–	–	0.038 ± 0.838	–	0.623	0.110	0.873	0.894
Wavelet-LHL_firstorder_Skewness (F3)	–	–	–0.059 ± 0.869	–	0.580	0.140	0.902	0.862
Lbp-3D-k_firstorder_MeanAbsoluteDeviation (F4)	–	–	0.180 ± 0.977	–	0.602	0.119	0.883	0.896
Lbp-3D-m2_glszm_GrayLevelVariance (F5)	–	–	–0.051 ± 0.879	–	0.655	0.008	0.866	0.907
DL_336 (F6)	–	–	0.213 ± 0.656	–	0.594	0.415	0.911	0.920
Wavelet-LLH_firstorder_10Percentile (F1)	–	–		–0.056 ± 0.891	0.647	0.040	0.857	0.863
Square_ngtdm_Busyness (F2)	–	–	–	–0.028 ± 0.555	0.687	0.006	0.881	0.897
DL_317 (F3)	–	–	–	0.082 ± 0.728	0.668	0.032	0.906	0.913
Wavelet-HHL_glcm_Imc1 (F4)	–	–	–	0.031 ± 0.662	0.664	0.024	0.923	0.927
Log-sigma-3-mm-3D_glszm_LargeAreaHighGrayLevel Emphasis (F5)	–	–	–	0.187 ± 0.837	0.653	0.154	0.893	0.8

## Discussion

The development of brain metastasis is a major limitation of life expectancy and quality of life for patients with malignant tumor due to neurological impairments (Quigley et al., 2013). Although early identification of the metastatic origin of brain metastasis is essential to make timely personalized treatment, it is still impossible for clinicians to make the decision by visual inspection of imaging data because there is no specific biomarker. Previous works have explored CAD-based techniques for predicting the primary tumor types (Ortiz-Ramon et al., 2017; Béresová et al., 2018), but both simply performed analysis across the whole tumor area and ignored variations of tumor cell distributions, which was pathobiologically learned as complex heterogeneity of brain metastasis (Siam et al., 2015).

In the present study, we explored a patient- and population-level clustering approach to partition the brain metastasis into two phenotypically consistent subregions, a marginal subregion (S1) and an inner subregion (S2), according to the combination of MRI pixel intensities and local entropy. The S1 has higher MRI pixel intensity and local entropy compared to S2. This was partially consistent with pathological findings that suggested the brain metastasis usually consists of a tumor active area and a necrotic area (Achrol et al., 2019). Before our study, there have been commonly used traditional intratumoral partition techniques, such as Otsu (Farhidzadeh et al., 2015) and k-means (Shang et al., 2021), which are based on the distribution of pixel intensities in the single slice of the tumor image, without considering variations and correlations between different tumor slices and individuals. The clustering approach explored in this study was better at reflecting tumor heterogeneity: (i) the patient-level clustering was applied slice by slice based on the integration of MRI pixel intensities and local entropy, which can better reflect the heterogeneity between different tumor slices; (ii) The population-level clustering was applied based on superpixels gathered from all patients, which can reflect variations and correlations between different individuals.

To reduce overfitting caused by the relatively small sample size, we proposed a RN-GAP net that used GAP to replace the traditional full-connect layers of the ResNet50 net. In the proposed RN-GAP, the Subnet 1 based on ResNet50 can effectively deal with the problem of gradient disappearance and degradation. Besides, the introduced GAP helps reduce the entire height and weight to a single vector by calculating the average of each feature map as an output point, and hence leads to dimensionality reduction without parameter optimization (Lin et al., 2013). Therefore, one advantage of the GAP is to avoid overfitting at this layer, and thus improving generalization abilities of the network. Another advantage is that the GAP strengthens the correspondence between feature maps and categories.

By combining both handcrafted and deep learning-based features, the proposed LR-NSCLC, LR-BC, LR-EGFR, and LR-HER2 achieved good prediction performance for predicting metastatic origins and EGFR/HER2 status, respectively. A total of twenty-three features were identified as the most predictive features to develop the four predictive models, sixteen were handcrafted features and seven were deep learning-based features. For predicting NSCLC and Breast cancer originated brain metastasis, our developed LR-NSCLC and LR-BC generated AUCs of 0.819–0.909, which were much higher than a previous study yielded AUCs of 0.63 and 0.61 (Kniep et al., 2019). This may be explainable since the previous study ignored intratumoral heterogeneity of the brain metastasis and lack of introducing deep learning techniques.

For predicting EGFR mutations, our proposed brain metastasis-based LR-EGFR (AUCs of 0.750–0.850) outperformed previously proposed traditional radiomics models based on primary lung cancer (AUCs ranged from 0.575 to 0.762) (Liu et al., 2016; Gevaert et al., 2017; Yuan et al., 2017; Zhang et al., 2018; Digumarthy et al., 2019; Pinheiro et al., 2020) and brain metastasis (AUCs ranged from 0.675 to 0.733) (Wang et al., 2021). This may be because previous studies only applied traditional handcrafted features to develop models. Our results suggested that the RN-GAP net is helpful to identify predictive deep learning-based features that provide complementary information to handcrafted features. For predicting HER2 status, our LR-HER2 generated AUCs of 0.803–0.900, which were much higher than previous studies based on primary breast cancer and generated AUCs ranging from 0.700 to 0.860 (Li et al., 2021; Niu et al., 2021; Zhou et al., 2021). Our findings suggested information correlated to the HER2 status can also be captured from brain metastasis. The identified most predictive features related to EGFR and HER2 status filtered handcrafted features and deep-learned features, which were hidden in high-dimensional spaces and cannot be identified by radiologists with naked eyes. In clinical practice, many patients whose primary tumors were surgically removed develop brain metastasis, for which cases, a non-invasive method using brain metastasis to early assess the metastatic origins and reflect EGFR or HER2 status is an important alternative for guiding personalized treatment plan (Witzel et al., 2016).

This study has several limitations. First, this is a retrospective study with a relatively small population. Validation of our findings with a larger multi-center sample is needed in our future work. Second, although our intratumoral partition method can effectively divide brain metastasis into various habitats and exhibited good predictive performance, our results can hardly be verified at the pathological level. Besides, there are still no uniform standards for intratumoral partition algorithms, for capturing tumor heterogeneity. Correlations between brain MRI-based morphological differences and corresponding histological microenvironment is warranted, for guiding the improvement of our intratumoral partition method. Third, considering different subtypes of lung cancer and breast cancer have different likelihoods of metastasizing to the brain (Soni et al., 2015), which were not studied in our work. Last, we included only the most common metastatic tumor types of NSCLC and breast cancer (incidence rates of 56–86% for brain metastasis). Some other metastatic tumor type (e.g., melanoma, incidence rates of 6–11% for brain metastasis) was not included due to the data collection challenge in our hospital, which should be investigated in our future studies.

## Conclusion

Our work indicated that subregional radiomics is of significant to predict metastatic origins and assess gene status based on brain metastasis. Our proposed handcrafted and deep learning feature combined logistic regression model may be clinically valuable to assist in early personalized treatment plan decisions.

## Data availability statement

The original contributions presented in this study are included in the article/Supplementary material, further inquiries can be directed to the corresponding author/s.

## Author contributions

JS, ZZ, HF, and XJ contributed to conception and design of the study. JL and YL organized the database. TJ and HA performed the statistical analysis. ZZ and XJ wrote the first draft of the manuscript. JS and XC wrote sections of the manuscript. All authors contributed to manuscript revision, read, and approved the submitted version.

## Footnotes

1
https://pypi.org/project/pyradiomics/

## Supplementary material

Supplementary Figure 1 Different CH values when the clusters k was set from 2 to 10.
