# Supplementary figures and images for: A deep learning approach with subregion partition in MRI image analysis for metastatic brain tumor
Source: Front Neuroinform. 2022 Aug 3;16:973698. doi: 10.3389/fninf.2022.973698 (PMC9382021; doi:10.3389/fninf.2022.973698)

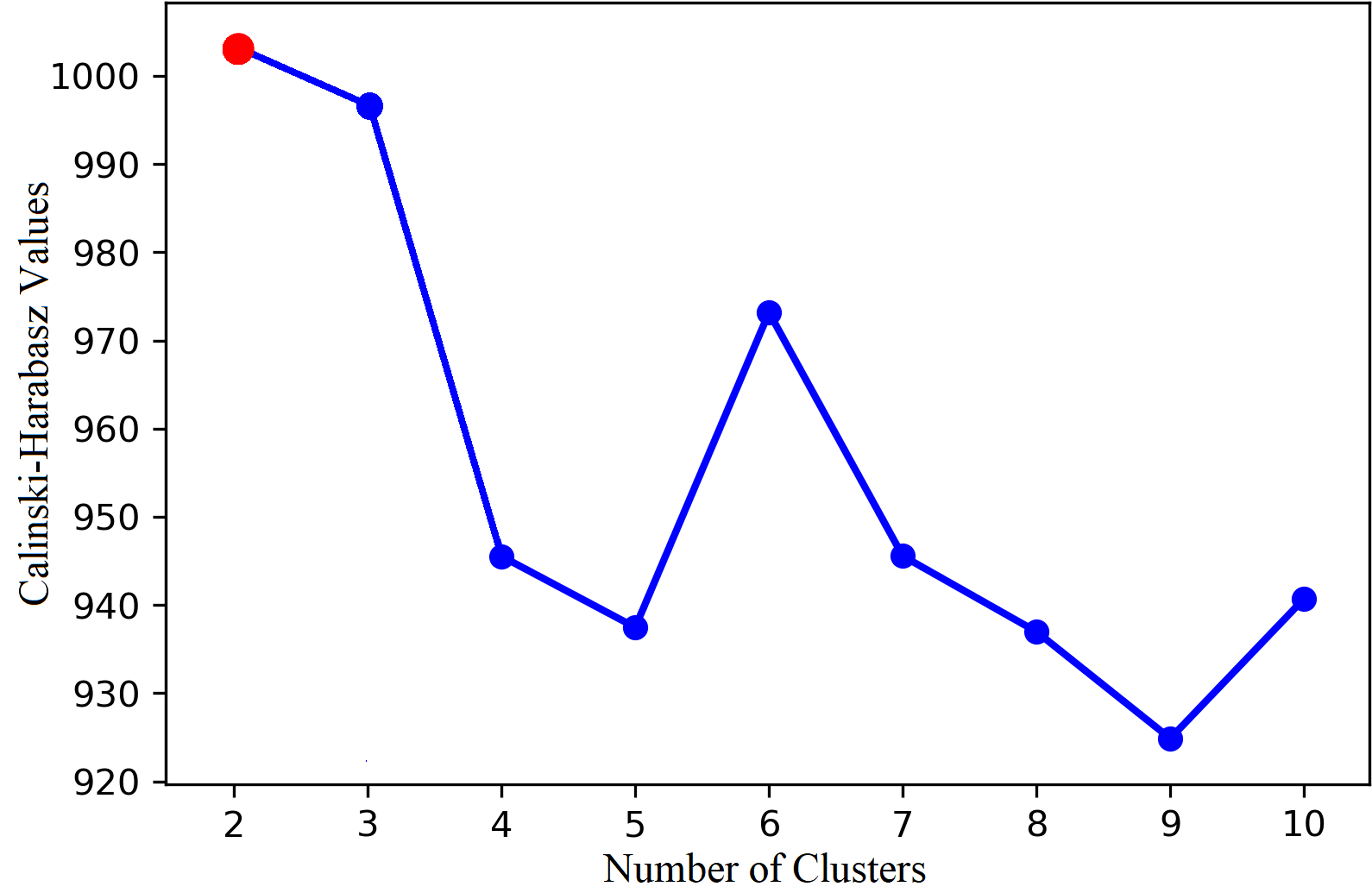

Supplement: Supplementary Figure 1 — Different CH values when the clusters k was set from 2 to 10. [file Image_1.TIF]
