# Supplementary material for: A deep learning approach with subregion partition in MRI image analysis for metastatic brain tumor
Source: Front Neuroinform. 2022 Aug 3;16:973698. doi: 10.3389/fninf.2022.973698 (PMC9382021; doi:10.3389/fninf.2022.973698)
Supplement: Supplementary file 2 [file Table_1.DOCX]

**Table S1**. The network architecture of ResNet50.

| **Layer name** | | | **Input size** | **Output size** |
| --- | --- | --- | --- | --- |
| Conv1 | | 7 × 7, 64 | 224 × 224 × 3 | 112 × 112 × 64 |
| MaxPooling | | 3 × 3 max pool | 112 × 112 × 64 | 56 × 56 × 64 |
| Res block 1 | Conv block | 1 × 1, 64 | 56 × 56 × 64 | 56 × 56 × 64 |
|  |  | 3 × 3, 64 | 56 × 56 × 64 | 56 × 56 × 64 |
|  |  | 1 × 1, 256 | 56 × 56 × 64 | 56 × 56 × 256 |
|  | Identity block | 1 × 1, 64 | 56 × 56 × 256 | 56 × 56 × 64 |
|  |  | 3 × 3, 64 | 56 × 56 × 64 | 56 × 56 × 64 |
|  |  | 1 × 1, 256 | 56 × 56 × 64 | 56 × 56 × 256 |
|  | Identity block | 1 × 1, 64 | 56 × 56 × 256 | 56 × 56 × 64 |
|  |  | 3 × 3, 64 | 56 × 56 × 64 | 56 × 56 × 64 |
|  |  | 1 × 1, 256 | 56 × 56 × 64 | 56 × 56 × 256 |
| Res block 2 | Conv block | 1 × 1, 128 | 56 × 56 × 256 | 28 × 28 × 128 |
|  |  | 3 × 3, 128 | 28 × 28 × 128 | 28 × 28 × 128 |
|  |  | 1 × 1, 512 | 28 × 28 × 128 | 28 × 28 × 512 |
|  | Identity block | 1 × 1, 128 | 28 × 28 × 512 | 28 × 28 × 128 |
|  |  | 3 × 3, 128 | 28 × 28 × 128 | 28 × 28 × 128 |
|  |  | 1 × 1, 512 | 28 × 28 × 128 | 28 × 28 × 512 |
|  | Identity block | 1 × 1, 128 | 28 × 28 × 512 | 28 × 28 × 128 |
|  |  | 3 × 3, 128 | 28 × 28 × 128 | 28 × 28 × 128 |
|  |  | 1 × 1, 512 | 28 × 28 × 128 | 28 × 28 × 512 |
|  | Identity block | 1 × 1, 128 | 28 × 28 × 512 | 28 × 28 × 128 |
|  |  | 3 × 3, 128 | 28 × 28 × 128 | 28 × 28 × 128 |
|  |  | 1 × 1, 512 | 28 × 28 × 128 | 28 × 28 × 512 |
| Res block 3 | Conv block | 1 × 1, 256 | 28 × 28 × 512 | 14 × 14 × 256 |
|  |  | 3 × 3, 256 | 14 × 14 × 256 | 14 × 14 × 256 |
|  |  | 1 × 1, 1024 | 14 × 14 × 256 | 14 × 14 × 1024 |
|  | Identity block | 1 × 1, 256 | 14 × 14 × 1024 | 14 × 14 × 256 |
|  |  | 3 × 3, 256 | 14 × 14 × 256 | 14 × 14 × 256 |
|  |  | 1 × 1, 1024 | 14 × 14 × 256 | 14 × 14 × 1024 |
|  | Identity block | 1 × 1, 256 | 14 × 14 × 1024 | 14 × 14 × 256 |
|  |  | 3 × 3, 256 | 14 × 14 × 256 | 14 × 14 × 256 |
|  |  | 1 × 1, 1024 | 14 × 14 × 256 | 14 × 14 × 1024 |
|  | Identity block | 1 × 1, 256 | 14 × 14 × 1024 | 14 × 14 × 256 |
|  |  | 3 × 3, 256 | 14 × 14 × 256 | 14 × 14 × 256 |
|  |  | 1 × 1, 1024 | 14 × 14 × 256 | 14 × 14 × 1024 |
|  | Identity block | 1 × 1, 256 | 14 × 14 × 1024 | 14 × 14 × 256 |
|  |  | 3 × 3, 256 | 14 × 14 × 256 | 14 × 14 × 256 |
|  |  | 1 × 1, 1024 | 14 × 14 × 256 | 14 × 14 × 1024 |
|  | Identity block | 1 × 1, 256 | 14 × 14 × 1024 | 14 × 14 × 256 |
|  |  | 3 × 3, 256 | 14 × 14 × 256 | 14 × 14 × 256 |
|  |  | 1 × 1, 1024 | 14 × 14 × 256 | 14 × 14 × 1024 |
|  | Identity block | 1 × 1, 256 | 14 × 14 × 1024 | 14 × 14 × 256 |
|  |  | 3 × 3, 256 | 14 × 14 × 256 | 14 × 14 × 256 |
|  |  | 1 × 1, 1024 | 14 × 14 × 256 | 14 × 14 × 1024 |
| Res block 4 | Conv block | 1 × 1, 512 | 14 × 14 × 1024 | 7 × 7 × 512 |
|  |  | 3 × 3, 512 | 7 × 7 × 512 | 7 × 7 × 512 |
|  |  | 1 × 1, 2048 | 7 × 7 × 512 | 7 × 7 × 2048 |
|  | Identity block | 1 × 1, 512 | 7 × 7 × 2048 | 7 × 7 × 512 |
|  |  | 3 × 3, 512 | 7 × 7 × 512 | 7 × 7 × 512 |
|  |  | 1 × 1, 2048 | 7 × 7 × 512 | 7 × 7 × 2048 |
|  | Identity block | 1 × 1, 512 | 7 × 7 × 2048 | 7 × 7 × 512 |
|  |  | 3 × 3, 512 | 7 × 7 × 512 | 7 × 7 × 512 |
|  |  | 1 × 1, 2048 | 7 × 7 × 512 | 7 × 7 × 2048 |
| AveragePooling | | | 7 × 7 × 2048 | 1 × 1 × 2048 |
| Flatten | | | 1 × 1 × 2048 | 2048 |
| fc 1000 | | | 2048 | 1000 |
| softmax | | | | |
