# Supplementary material for: A deep learning approach with subregion partition in MRI image analysis for metastatic brain tumor
Source: Front Neuroinform. 2022 Aug 3;16:973698. doi: 10.3389/fninf.2022.973698 (PMC9382021; doi:10.3389/fninf.2022.973698)
Supplement: Supplementary file 3 [file Table_2.DOCX]

**Table S2**. Settings of parameters of the pre-processing of MR images.

| **Parameter** | **Value** |
| --- | --- |
| normalize | true |
| normalizeScale | 100 |
| interpolator | B-spline |
| binWidth | 10 |
